# Repurposing Albendazole: new potential as a chemotherapeutic agent with preferential activity against HPV-negative head and neck squamous cell cancer

**DOI:** 10.18632/oncotarget.17292

**Published:** 2017-04-20

**Authors:** Farhad Ghasemi, Morgan Black, Frederick Vizeacoumar, Nicole Pinto, Kara M. Ruicci, Carson Cao Son Huu Le, Matthew R. Lowerison, Hon Sing Leong, John Yoo, Kevin Fung, Danielle MacNeil, David A. Palma, Eric Winquist, Joe S. Mymryk, Paul C. Boutros, Alessandro Datti, John W. Barrett, Anthony C. Nichols

**Affiliations:** ^1^ Department of Otolaryngology, London Health Sciences Centre, London, Ontario, Canada; ^2^ Department of Oncology, London Health Sciences Centre, London, Ontario, Canada; ^3^ Lunenfeld Tanenbaum Research Institute, Mount Sinai Hospital, Toronto, Ontario, Canada; ^4^ Department of Surgery, Schulich School of Medicine, Western University, London, Ontario, Canada; ^5^ Translational Prostate Cancer Research Laboratory, Lawson Health Research Institute, London, Ontario, Canada; ^6^ Department of Medical Biophysics, Western University, London, Ontario, Canada; ^7^ Robarts Research Institute, Western University, London, Ontario, Canada; ^8^ Department of Microbiology and Immunology, University of Western Ontario, London, Ontario, Canada; ^9^ Informatics and Biocomputing Program, Ontario Institute of Cancer Research, MaRS Centre, Toronto, Ontario, Canada; ^10^ Department of Medical Biophysics, University of Toronto, Toronto, Ontario, Canada

**Keywords:** head and neck cancer, human papillomavirus, anti-helminthic, cell cycle arrest

## Abstract

Albendazole is an anti-helminthic drug that has been shown to exhibit anti-cancer properties, however its activity in head and neck squamous cell cancer (HNSCC) was unknown. Using a series of *in vitro* assays, we assessed the ability of albendazole to inhibit proliferation in 20 HNSCC cell lines across a range of albendazole doses (1 nM–10 μM). Cell lines that responded to treatment were further examined for cell death, inhibition of migration and cell cycle arrest. Thirteen of fourteen human papillomavirus-negative HNSCC cell lines responded to albendazole, with an average IC_50_ of 152 nM. In contrast, only 3 of 6 human papillomavirus-positive HNSCC cell lines responded. Albendazole treatment resulted in apoptosis, inhibition of cell migration, cell cycle arrest in the G2/M phase and altered tubulin distribution. Normal control cells were not measurably affected by any dose tested. This study indicates that albendazole acts to inhibit the proliferation of human papillomavirus-negative HNSCC cell lines and thus warrants further study as a potential chemotherapeutic agent for patients suffering from head and neck cancer.

## INTRODUCTION

Head and neck cancer is the 6th most common cancer diagnosis worldwide, with a reported annual incidence of over 550,000 cases [1]. Although tobacco and alcohol consumption are recognized as the two main risk factors for the development of head and neck squamous cell cancer (HNSCC), a subset of the tumours are associated with human papillomavirus (HPV) infection and viral oncogene expression. There has been considerable focus on the management of this HPV-positive disease due to the dramatic rise of incidence in patients that are typically younger in age and healthier [2]. The favorable survival outcomes in the HPV-positive patients has led to significant efforts to de-intensify their treatment [3]. In contrast, patients with HPV-negative disease make up a large portion of HNSCC (∼40–60%) and experience markedly poorer survival outcomes relative to those that are HPV-positive [4, 5]. There is a critical need to identify novel therapeutics for patients with classic risk factors (smoking and drinking) that lead to HPV-negative HNSCC disease.

Albendazole is a benzimidazole carbamate with an extensive history of safe and routine use in both humans and animals to eliminate parasitic worms [6, 7]. The anti-helminthic ability of albendazole and other drugs of the benzimidazole class has been primarily attributed to their tubulin binding capacity, resulting in de-polymerization and cell-cycle arrest in nematodes [8]. The ability of benzimidazoles to inhibit microtubule polymerization has led to investigation of their anti-cancer potential in melanoma, ovarian, colorectal and hepatocellular cancer models [9–12]. In addition to disrupting tubulin assembly and causing cell cycle arrest in cancer cells, initial studies demonstrated that this drug significantly reduced both VEGF and HIF-1α activity and inhibited angiogenesis [13, 14]. Albendazole has also been recognized as a radiosensitizer in melanoma and small-cell lung cancer models [9].

Here we demonstrate that albendazole markedly reduces HNSCC cell-growth, with preferential activity in HPV-negative cell lines. We further show that albendazole can cause cell death and apoptosis, reduce cellular migration, induce cell-cycle arrest, and alter tubulin polymerization within *in vitro* HNSCC experimental models.

## RESULTS

### Albendazole inhibited HNSCC cell line proliferation, with preferential activity in HPV-negative cell lines

**Figure 1:**
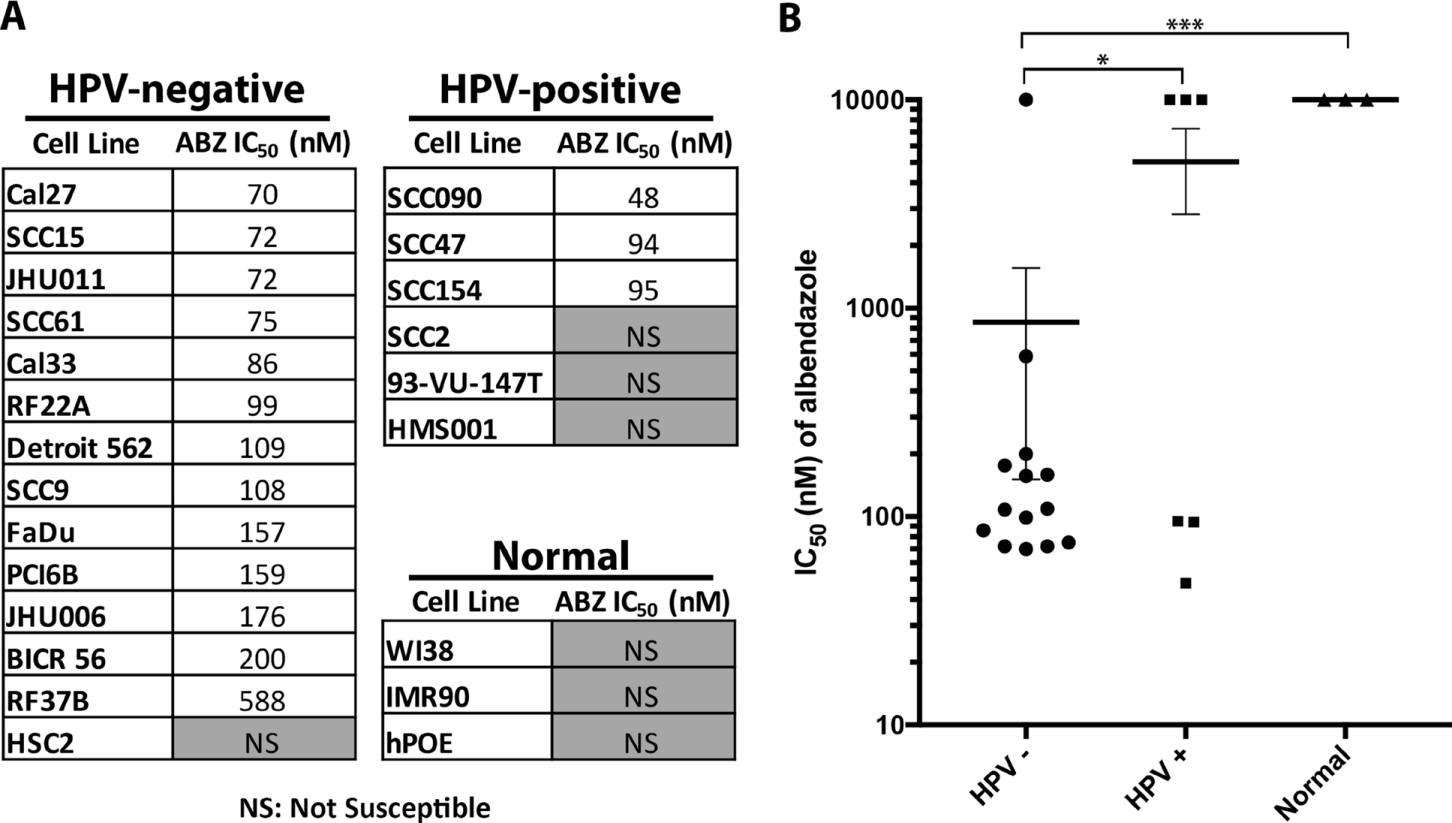
Albendazole inhibited the cell growth of HNSCC cell lines, with preferential activity in HPV-negative cell lines (**A**) HPV-negative, HPV-positive and normal cell lines were tested against increasing doses of albendazole in order to calculate IC_50_ values. Cell lines that did not reach 50% viability at the maximum dose (10 μM) are denoted as NS (not susceptible). (**B**) Mean potency of albendazole for HPV-negative, HPV-positive and normal cell lines. Cell lines that did not reach IC_50_ were assigned a value of 10 μM (highest concentration tested) for statistical analysis. This potency trend suggests that albendazole demonstrates preferential activity in HPV-negative cell lines (unpaired *t*-test; *p <* 0.05). Error bars reflect standard error, *p < 0.05 and ***p < 0.001.

We tested 20 HNSCC cell lines (14 HPV-negative and 6 HPV-positive) with a range of albendazole doses (1 nM–10 μM) to calculate half-maximal inhibitory concentration (IC_50_) values. Cells that did not reach 50% viability relative to the control were considered non-susceptible in the concentration range tested, and were assigned an IC_50_ value of 10 μM (maximum dose tested) for further analyses. Albendazole inhibited growth in 13 of 14 (93%) HPV-negative cell lines at the tested doses when compared to the untreated cells (Figure 1A). In contrast, only 3 of 6 (50%) HPV-positive cell lines exhibited reduced growth in response to albendazole treatment at the doses tested (Figure 1A). HPV-negative cell lines had lower mean IC_50_ values compared to the HPV-positive HNSCC lines (*p* = 0.03, Figure 1B). Three normal cell lines were also treated with albendazole and none reached an IC_50_ in the dose range tested, confirming the specificity of albendazole for cancer cell lines.

### Albendazole treatment caused cell death and apoptosis in the susceptible cell lines

**Figure 2:**
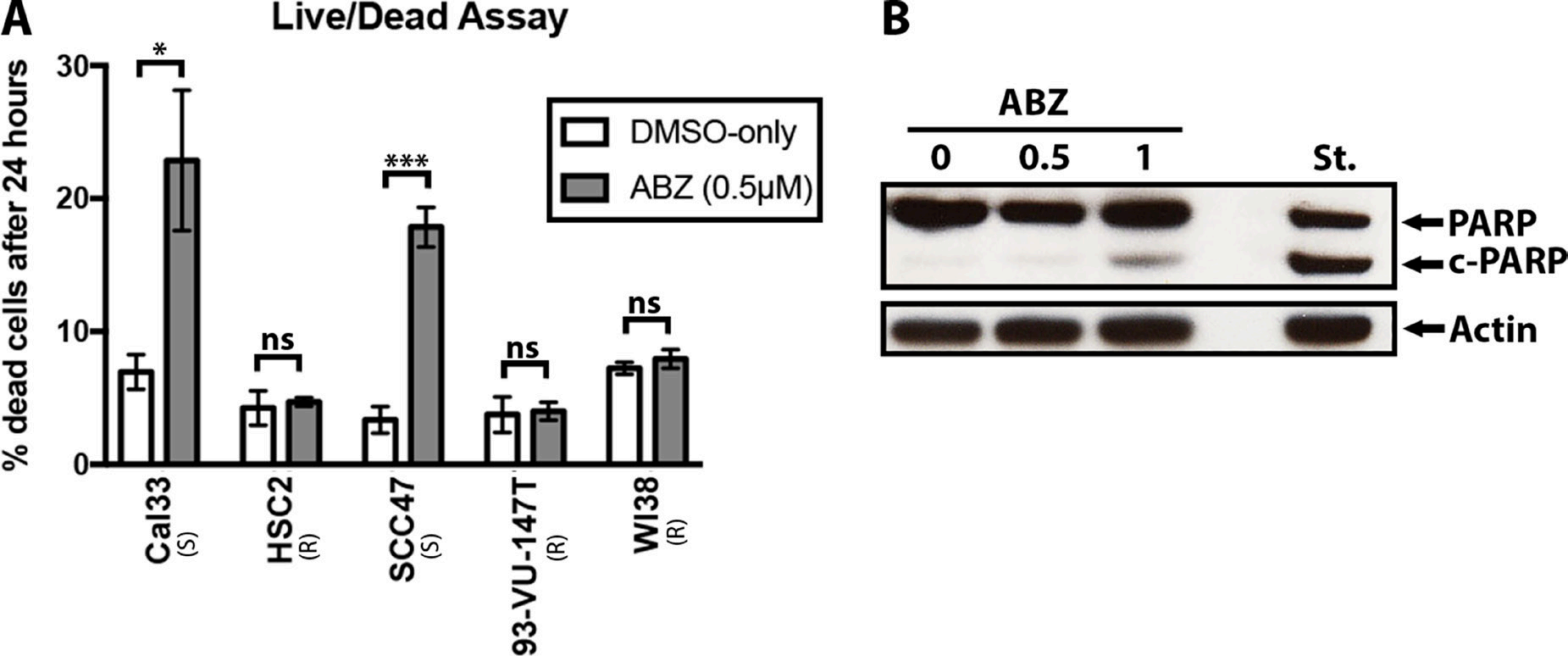
Albendazole caused cell death and apoptosis (**A**) Live/dead assays were performed with Cal33, HSC2, SCC47, 93-VU-147T and WI38 cell samples that were exposed to vehicle (DMSO-only) or 0.5 μM of albendazole (ABZ) for 24 hours (four replicates per treatment). Albendazole treatment significantly increased the percentage of dead cells in susceptible cell lines (Cal33 and SCC47; paired *t*-test, *p* = 0.026 and *p <* 0.001 respectively), but yielded insignificant changes in HSC2, 93-VU-147T and WI38 cell lines. “(S)” marks the susceptible, and “(R)” marks the non-susceptible cell lines by IC50 analysis. Error bars show standard error, *p < 0.05 and **p < 0.01. (**B**) Cal33 cells were exposed to vehicle (DMSO-only), 0.5 μM or 1 μM ABZ for 24 hours, and immunoblotted for PARP. Staurosporine (St.) treatment was used as a positive control for apoptosis. Presence of cleaved PARP (c-PARP) at 1 μM albendazole treatment suggested that apoptosis was involved in cell death.

To measure the effect of albendazole on cell health, we performed live/dead assays. A significant increase in the frequency of cell death was measured after the addition of albendazole in the susceptible cell lines (Figure 2A). This observation was true regardless of whether the cells were HPV-positive or HPV-negative. Statistical analysis (paired *t*-test) showed that albendazole caused a significant increase in the percent of dead cells within susceptible (Cal33 *p* = 0.028 and SCC47 *p* = 0.0006) cell lines, but no significant changes in the non-susceptible (HSC2 *p* = 0.75, 93-VU-147T *p* = 0.56) and normal (WI38 *p* = 0.53) cell lines. Thus, the ability of albendazole to induce cell death is fully concordant with the reduction in cell proliferation described above.

Based on previous studies, we hypothesized that the drug-induced cell death may involve apoptosis [9]. Immunoblotting with an anti-PARP antibody demonstrated that albendazole treatment induced partial cleavage of PARP in a dose dependent manner (Figure 2B). Weak PARP cleavage was visible starting at 0.5 uM and increased as the dose was further escalated (Figure 2B).

### Albendazole reduced cell migration in the susceptible HNSCC cell lines

**Figure 3:**
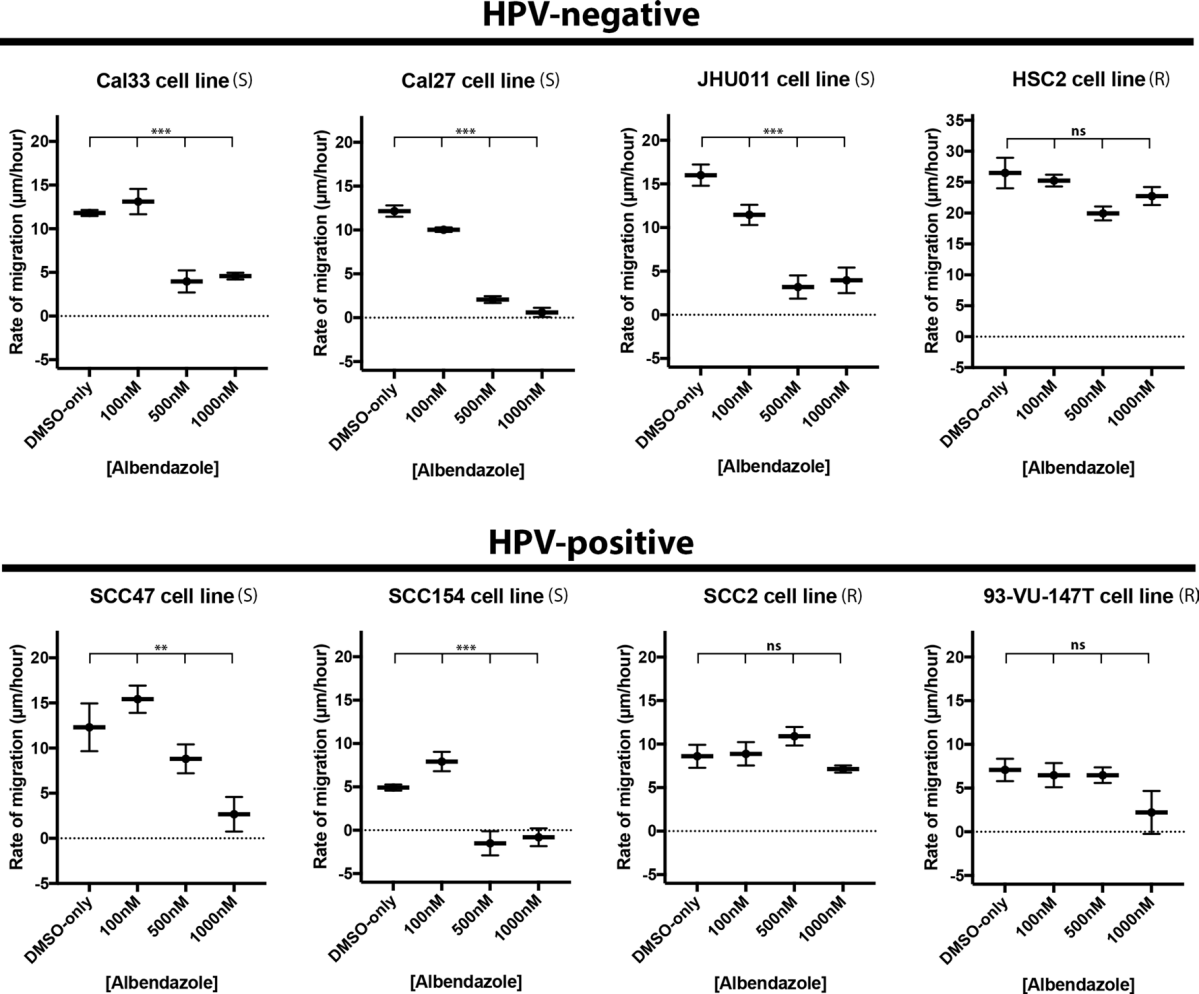
Albendazole impaired cell migration Scratch assays were performed with 4 HPV-positive and 4 HPV-negative cell lines, exposed to various concentrations of albendazole, and imaged at 0, 2, 5, 8, and 12 hours post-treatment. Linear regression analysis was used to calculate the rate of growth (in μm/hour). Albendazole significantly reduced the rate of cell migration in cell lines that were susceptible in IC50 analysis, and led to no significant changes in migration rate in the non-susceptible cell lines (ANCOVA analysis, ns = not significant, **p < 0.01, ***p < 0.001). “(S)” marks the susceptible, and “(R)” marks the non-susceptible cell lines by IC50 analysis. Error bars represent standard error.

In an effort to observe the effect of albendazole on cell migration, we performed scratch assays in 4 HPV-negative and 4 HPV-positive HNSCC cell lines (Figure 3). Albendazole treatment significantly reduced the rate of migration in the cell lines considered susceptible by the cell proliferation studies (Cal33 *p <* 0.0001, Cal27 *p <* 0.0001, JHU011 *p <* 0.0001, SCC47 *p* = 0.0039, SCC154 *p <* 0.0001). This effect was generally observed at doses at or greater than 0.5 μM and was independent of HPV status (Figure 3). In contrast, non-susceptible cell lines did not respond with a significant change in the migration rate to doses of albendazole as high as 1 μM (*p* > 0.05 in HSC2, SCC2 and 93-VU-147T).

### Albendazole blocked cell cycle progression

**Figure 4:**
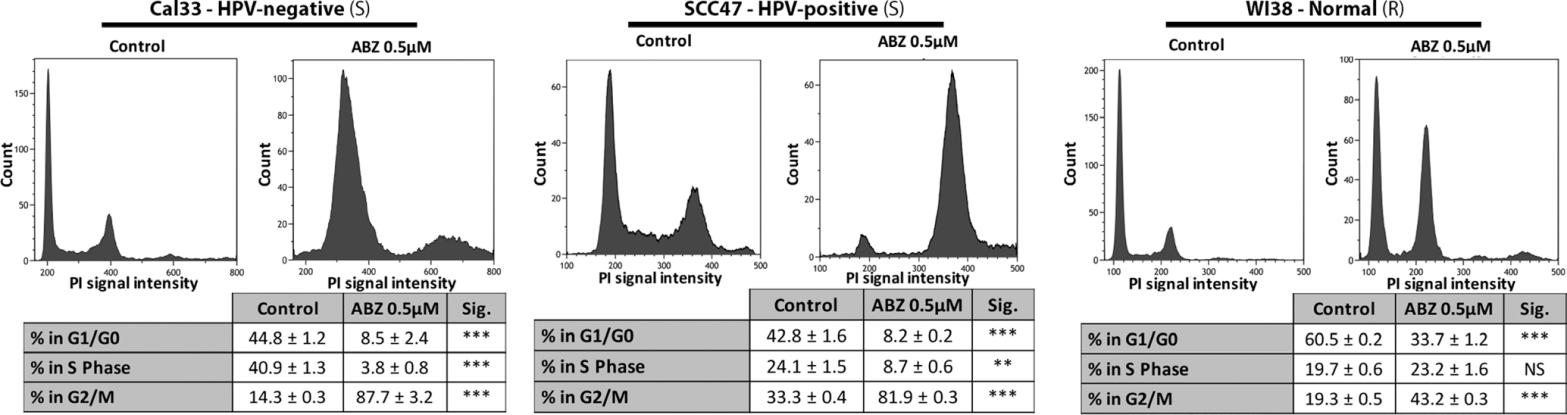
Albendazole induced cell cycle arrest at G2/M phase Cal33 (HPV-negative), SCC47 (HPV-positive) and WI38 (normal) cells were exposed to vehicle (DMSO-only) or 0.5 μM albendazole (ABZ) for 24 hours (3 replicates per treatment, ± standard error is shown). BrdU and PI staining were carried out prior to flow cytometry analysis. “(S)” marks cells lines susceptible, and “(R)” marks cell lines not susceptible to albendazole in IC50 analysis. *p < 0.05, **p < 0.01, ***p < 0.001, and NS = not significant.

To determine how the cell cycle was being affected by albendazole treatment, we performed flow cytometry analysis. Flow cytometry indicated that albendazole treatment altered the distribution of cells in various phases of the cell-cycle (Figure 4). In all cell lines tested, albendazole resulted in the accumulation of cells in the G2/M phase of the cell cycle. The average increase in the frequency of G2/M phase cells following treatment with albendazole was significantly less in the normal WI38 cell line than Cal33 (unpaired *t*-test, *p <* 0.0001) or SCC47 (unpaired *t*-test, *p <* 0.0001) cell lines. We also observed a reduced proportion of S phase cells in the HNSCC cell lines, which further supports the hypothesis that albendazole acts to inhibit cell proliferation.

### Albendazole altered cellular tubulin distribution

**Figure 5:**
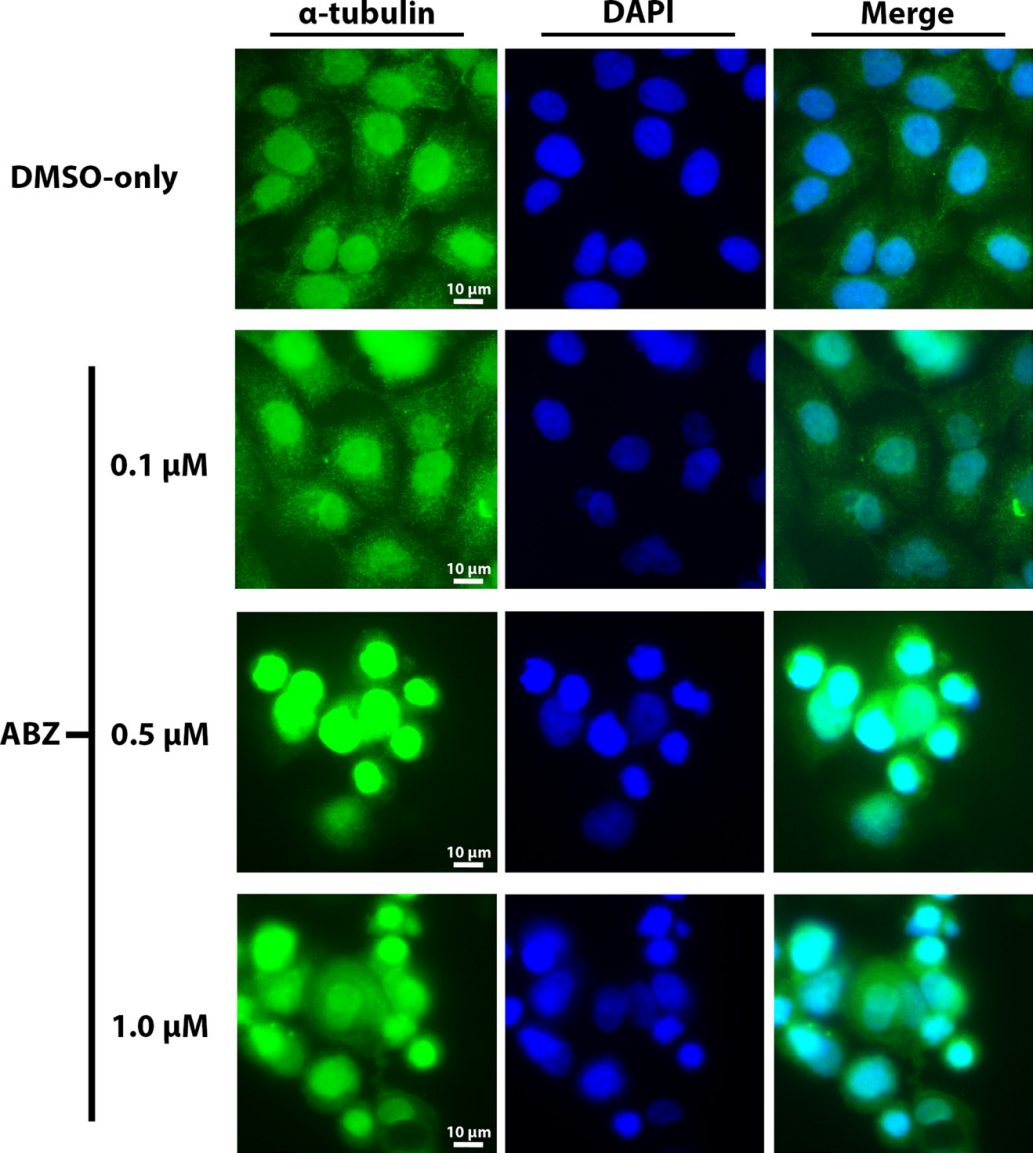
Dose-dependent disruption in the distribution of α-tubulin following exposure to albendazole Experimental treatments included vehicle (DMSO), 0.1 μM, 0.5 μM aornd 1 μM albendazole for 24 hours. α-tubulin was visualized using immunofluorescence (green) and nuclei were stained with DAPI (blue). 40x fluorescence microscopy was used to assess changes in cellular morphology and tubulin distribution.

Based on the known ability of albendazole to disrupt tubulin assembly, we examined α-tubulin distribution within Cal33 cells in response to treatment with various doses of albendazole. Visualization of α-tubulin distribution within Cal33 cells by indirect immunofluorescence revealed a dose-dependent change in tubulin distribution and alteration of the tubulin assembly (Figure 5). Specifically, treatment with just 0.1 μM albendazole caused a slightly increased concentration of tubulin around the nuclei as compared to the normal cytoplasmic distribution of α-tubulin in the vehicle (DMSO-only) treated cells. Tubulin distribution became progressively perinuclear at higher drug concentrations. Indeed, at higher albendazole doses of 0.5 μM and 1 μM there is a distinct absence of ordinary cytoplasmic tubulin distribution, with the appearance of concentric clumping of tubulin around the nuclei and heavy nuclear staining.

## DISCUSSION

The development of targeted cancer therapies based on personalized medicine has been considered by many to be the future of cancer treatment. One consequence of the huge development and testing costs for these treatments has been a rapid and economically unsustainable increase in the cost of new cancer drugs [15]. As an attractive alternative solution, repurposing the large arsenal of existing approved, non-cancer drugs for cancer treatment has become the focus of intense investigation [16].

Albendazole is an approved anti-parasitic drug with an extensive history of safe and routine use in humans since it was first approved in 1982 [6, 7]. Due to the accumulating evidence that this drug may have potent growth inhibitory effect on multiple types of cancer cells [8–12], we decided to investigate albendazole for its effects on head and neck cancer cells.

Albendazole had a potent anti-proliferative effect in our panel of HNSCC cell lines, and we demonstrate that it has preferential activity against HPV-negative lines. Growth inhibition was accompanied by increased cell death in all drug sensitive cell lines. Cell death was likely mediated by increased apoptosis, based on an increased level of PARP cleavage. As the prognosis for patients with HPV-negative tumors is much worse than for HPV-positive cancers [4], the improved activity of albendazole against HPV-negative cell lines has important clinical ramifications. The reduced effectiveness of albendazole in HPV-positive HNSCC cell lines may be due to an HPV-related inhibition of apoptosis [17]. Further work is needed to elucidate the mechanisms underlying the differential treatment responses in HPV-positive and negative cell lines.

Consistent with previous studies in other cancer types, flow cytometry analysis demonstrated that albendazole treatment arrested HNSCC cells in the G2/M phase. This may result, at least in part from albendazole's known ability to inhibit tubulin polymerization [18], which is essential for mitosis. We confirmed that albendazole treatment dramatically altered the appearance and distribution of tubulin in HNSCC cells.

The observation that albendazole triggers an arrest in the G2/M stage of the cell cycle is intriguing, as different cell cycle phases are associated with sensitivity to radiotherapy, with the impact of radiation maximized during the G2/M phase of the cycle [19]. Investigations with small-cell lung cancer and melanoma cells have demonstrated that albendazole and radiation can act in synergy to inhibit cancer cell growth [9]. These findings similarly suggest the potential application of albendazole as a radiosensitizer in HNSCC treatment.

The routine use of the benzimidazoles in humans for control of infectious diseases highlights their safety and clinical applicability. However, the low aqueous solubility and bioavailability of benzimidazoles is a potential limiting factor in the use of these drugs as cancer therapeutics [20–22]. Investigations with intravenous delivery of the albendazole, nanoparticle assemblies and solid dispersion techniques offer potential solutions to increase the absorbance and plasma concentration of albendazole [23–27]. Further research is required to assess the response of head and neck cancer cells to these drug delivery methods.

In conclusion, albendazole has shown strong potential as a chemotherapeutic agent with high potency against HNSCC cell lines, particularly in the HPV-negative subset. We have shown that albendazole can cause cell death through activation of apoptosis, initiation of cell-cycle arrest and alteration of tubulin distribution *in vitro* in HNSCC cell line models, collectively highlighting its multi-faceted ability to control the growth of these cancer cells.

## MATERIALS AND METHODS

### Reagents

Albendazole was obtained from Selleck Chemicals^®^. Albendazole was dissolved in dimethyl sulfoxide (DMSO) at a concentration of 10 mM to create a stock solution and stored at −80°C.

### Cell cultures

Human WI38, IMR90, HSC2 and primary oral epithelial cells (hPOE; Abm Inc.) were grown in EMEM (Wisent) supplemented with 10% fetal bovine serum (FBS; Wisent), penicillin (100 IU/mL; Invitrogen) and streptomycin (100 μg/mL; Invitrogen). Detroit 562, Cal27, SCC9, FaDu, SCC15 were purchased from ATCC. RF37B and RF22A were a kind gift of Dr. Robert Ferris (University of Pittsburgh), UPCI:SCC090 and UPCI:SCC154 were obtained from Dr. Suzanne Gollin (University of Pittsburgh), PCI6B (University of Pittsburgh), HMS001 (a gift of Dr. James Rocco, Harvard Medical School), JHU011 (Johns Hopkins University), JHU006 (Johns Hopkins University), 93-VU-147T gift of Dr. Johan de Winter, VU Medical Center, Amsterdam, and SCC61 were a gift of Dr. Wendell Yarborough, Yale University. Most HNSCC cell lines and were grown in DMEM/F12 (Wisent) supplemented with 10% fetal bovine serum (FBS; GIBCO), penicillin (100 IU/mL; Invitrogen) and streptomycin (100 μg/mL; Invitrogen). Cal33 cells were grown in DMEM (Wisent) supplemented with 10% heat-inactivated fetal bovine serum (FBS; GIBCO), penicillin (100 IU/mL; Invitrogen) and streptomycin (100 μg/mL; Invitrogen). BICR56 cells were grown in DMEM (Wisent) supplemented with 10% fetal bovine serum (FBS; Wisent), penicillin (100 IU/mL; Wisent), streptomycin (100 μg/mL; Wisent), hydrocortisone (0.4μg/ml, Wisent) and glutamine (2 mM, Wisent). Cells were incubated at 37°C and 5% CO_2_.

### Drug potency assay

Cell lines were seeded in 96-well plates at 3000 cells/well. 24 hours later, albendazole was added in a concentration series of 1, 3, 10, 30, 100, 300, 1000, 3000 and 10000 nM. Cells were exposed to drug for 72 hours before assessing potency. For each drug concentration, three replicates were carried out per cell line. Cell viability was measured indirectly using the PrestoBlue^®^ Reagent (Thermofisher Scientific). To calculate the half-maximal inhibitory concentration (IC_50_) value for each cell line, the media alone (blank) was subtracted from the Relative Fluorescence Unit (RFU) measures of each replicate to account for the background signal. Normalized RFUs of the drug-treated replicates were calculated as a percentage of the mean RFU of the vehicle control (DMSO-only) treatment replicates. IC_50_ values, defined as the concentration at which the normalized RFU reached 50%, were calculated by non-linear regression (Prism^®^ 7 Graphpad Software, Inc). Only cell lines that dropped below 50% normalized RFU within the dose range tested were considered to be substantially affected by drug. An unpaired *t*-test was used to assess the significance of the differences in IC_50_ values between HPV-positive and HPV-negative cell lines.

### Live/dead assay

Cal33, HSC2, SCC47, 93-VU-147T and WI38 cell lines were seeded in 60 mm plates at a density of 100,000 cells/mL. Twenty-four hours later, samples were treated with either vehicle control (DMSO) or albendazole (500 nM). Four replicates of each treatment were used. Plates were incubated at 37°C for 24 hours. Samples were trypsinized, and resuspended in media. For each replicate, 10 μL resuspended cells were mixed with Trypan blue (Sigma-Aldrich) at a 1:1 ratio, allowed to incubate for 10 minutes, and placed on a hemocytometer for imaging under brightfield microscopy. The ratio of live:dead cells was then calculated for each plate. Two readings of each sample were performed. Statistical significance was determined using a paired *t*-test as DMSO-only and drug-treated replicate readings were done in pairs.

### Western blotting

Cal33 cells were treated with DMSO, 500 nM or 1 μM albendazole for 24 hours. As a positive control for apoptosis, Cal33 cells were treated with 1μM staurosporin (Selleck Chemicals) for 4 hours. PARP primary antibody (Genetex Inc., cat. GTX100573), was administered according to manufacturer recommendations. Actin levels were detected using a rabbit anti-actin primary antibody (Sigma-Aldrich, cat. A2066), and the same secondary antibody (G-anti-R) and detection method as previously noted. Western blot image is presented as scanned.

### Scratch assay

Cells were seeded and allowed to grow in 6-well plates until a confluent monolayer was formed. Each well was then scratched across using a P1000 pipette tip. Media was aspirated and the wells were washed twice using PBS. Media (2 mL) with the drug concentration of choice was added to each well. Treatment groups included DMSO-only, 100 nM, 500 nM, or 1 μM albendazole. Regions of the scratch to be imaged were then marked. Plates were immediately imaged under a 4× objective to calculate the initial distance between the two edges of scratch in each well. This served as the 0 hour control. Cells were then incubated at 37°C and imaging was repeated at 2, 5, 8 and 12 hours. The distance of migration of the edge was measured (μm) at 10 points for each image. Rate of migration (μm per hour) and standard error calculated for each treatment using Prism^®^ 7. Statistical analysis was done through ANCOVA analysis in the Prism^®^ 7 software.

### Flow cytometry

To determine the potential effects of albendazole on cell cycle, we treated Cal33, SCC47 and WI38 cells with DMSO-only and 0.5 μM of albendazole for 24 hours. Three replicates of each treatment were prepared. Cell proliferation labeling reagent (5-bromo-2′-deoxyuridine and 5-fluoro-2′-deoxyuridine) (GE Healthcare, cat. RPN201) was added to the media and cultures were incubated for 2 hours prior to harvesting. Cells were trypsinized, resuspended in media and washed 3 times with PBS. 95% Ethanol was used to fix cells. Cells were exposed to 2N HCL/0.5% Triton X-100 and then 0.1 M NaB_4_O_7_ for permeabilization. Mouse anti-BrdU primary antibody (1:50, BD Biosciences lot. 347580) and FITC-conjugated horse anti-mouse secondary antibody (1:25, Vector Laboratories cat. FI-2000) were added respectively, and incubated for 30 minutes per step, at room temperature and protected from light. Cells were then collected and resuspended in propidium iodide (PI;10 mg/mL) and RNase A (0.25 mg/mL, Bioshop Canada Inc., cat. RNA675), and incubated overnight at 4°C. To remove cell aggregates, cell suspensions were passed through cell strainers (Falcon^®^ Corning Science Ref. 352235). Flow cytometry analysis was then performed (Beckman Coulter Inc, Cytomics^TM^ FC500). Unpaired *t*-test statistical analyses were performed for all of the comparisons.

### Immunofluorescence

Cal33 cells were seeded and grown on glass coverslips and exposed to DMSO-only, 100 nM, 500 nM or 1 μM albendazole treatments for 24 hours. Three replicates of each treatment were prepared. Cells were fixed to the coverslips using 100% methanol (pre-chilled at −20°C). Samples were blocked using 5% normal donkey serum (Wisent) in PBS. Cells were then incubated for 1 hour with α-tubulin primary antibody (1:50, Cell Signaling Technology Cat. ID 2125) at room temperature, then washed extensively with PBS. Alexa Fluor^®^ 488 goat anti-rabbit IgG secondary antibody (1:1000, Molecular Probes, Inc.A-11008) was added to each slide and then incubated in the dark for 60 minutes. DAPI (Molecular Probes, Inc., 30 nM) was used for nuclear staining. Images were obtained under 40x magnification using an Olympus Provis AX70 microscope.
